# LepEU: A Consortium to Study the Population Genomics of Butterflies and Moths Across Europe

**DOI:** 10.1111/eva.70246

**Published:** 2026-05-12

**Authors:** Patrícia Beldade, Marta Vila, Saad Arif, Audrey Bourgois, Jon Bridle, Laurence Després, Marianne Elias, Marianne Espeland, Zdenek F. Fric, Pável Matos‐Maraví, Kiwoong Nam, Riccardo Poloni, Mar Repullés, Marjo Saastamoinen, Rachel A. Steward, Kalle Tunström, Başak Ulaşli, Teo Valentino, Arne Weinhold, Martin Wiemers, Richard I. Bailey, Mark Blaxter, Vlad Dincă, Kay Lucek, Joana I. Meier, Matthew E. Nielsen, Constantí Stefanescu, Charlotte J. Wright, LepEU Community, Mathieu Joron, Christopher W. Wheat

**Affiliations:** ^1^ Center for Ecology, Evolution and Environmental Changes (cE3c) & Global Change and Sustainability Institute (CHANGE), Faculty of Sciences University of Lisbon Lisbon Portugal; ^2^ Department of Biology, Faculty of Sciences University of A Coruna A Coruna Spain; ^3^ Department of Biological and Medical Sciences, Centre for Functional Genomics Oxford Brookes University Oxford UK; ^4^ Centre d'Ecologie Fonctionnelle et Evolutive, CEFE Univ Montpellier, CNRS, EPHE, IRD Montpellier France; ^5^ Department of Genetics, Evolution and Environment University College London London UK; ^6^ Laboratoire d'Ecologie Alpine Université Grenoble Alpes, CNRS Grenoble France; ^7^ Institut de Systématique, Évolution, Biodiversité, Muséum national d'Histoire naturelle CNRS, Sorbonne Université, EPHE‐PSL, Université des Antilles Paris France; ^8^ Centre Interdisciplinaire de Recherche en Biologie Collège de France, Université PSL, CNRS Paris France; ^9^ Leibniz Institute for the Analysis of Biodiversity Change (LIB) Museum Koenig Bonn Germany; ^10^ Biology Centre CAS Institute of Entomology České Budějovice Czech Republic; ^11^ DGIMI, INRAE University of Montpellier Montpellier France; ^12^ Faculty of Science University of South Bohemia České Budějovice Czech Republic; ^13^ Department of Organismal and Evolutionary Biology, Faculty of Biological and Environmental Sciences University of Helsinki Helsinki Finland; ^14^ Department of Biology Lund University Lund Sweden; ^15^ Department of Plant Protection, Faculty of Agriculture Hatay Mustafa Kemal University Antakya Hatay Turkey; ^16^ Biodiversity Genomics Laboratory Institute of Biology, University of Neuchâtel Neuchâtel Switzerland; ^17^ Cellular and Organismic Networks, Faculty of Biology Ludwig‐Maximilians‐Universität München Munich Germany; ^18^ Senckenberg – Leibniz Institution for Biodiversity and Earth System Research Senckenberg German Entomological Institute Müncheberg Germany; ^19^ Department of Ecology and Vertebrate Zoology, Faculty of Biology and Environmental Protection University of Lodz Łódź Poland; ^20^ Tree of Life Programme Wellcome Sanger Institute Cambridgeshire UK; ^21^ “Grigore Antipa” National Museum of Natural History Bucharest Romania; ^22^ Department of Zoology University of Cambridge Cambridge UK; ^23^ Institute of Ecology University of Bremen Bremen Germany; ^24^ Natural Sciences Museum of Granollers Barcelona Spain; ^25^ Department of Zoology Stockholm University Stockholm Sweden

**Keywords:** biodiversity management, clinal variation, conservation genomics, demographic history, insect decline, lepidoptera, local adaptation, phylogeography, population genomics, population structure

## Abstract

LepEU, the European Lepidopteran Population Genomics Consortium, was launched in 2023 to coordinate continental‐scale collections and generate population‐level genomic data for butterflies and moths across Europe. Its whole‐genome resequencing strategy takes advantage of the growing availability of reference genomes for Lepidoptera. LepEU supports scalable, standardized sampling and sequencing to quantify genetic diversity and population resilience. These data will allow addressing long‐standing questions about geographic patterns of biodiversity in this iconic clade for evolutionary ecology, while also filling critical gaps in biodiversity monitoring and conservation. The consortium's first in‐person workshop was held in Montpellier (France) in 2025 and was supported by the COST Action 10kLepGenomes. It gathered 23 researchers from 10 countries, including experts in Lepidoptera ecology, evolution, and population genomics. Building on 2 years of video conferencing, the workshop enabled participants to draw a roadmap for trans‐continental standardized sampling, isolate DNA of specimens collected during 2024, outline key research questions for LepEU, and discuss an outreach strategy to engage additional stakeholders. LepEU is coordinating with other international networks and consortia to develop a collaborative platform for tracking European lepidopteran biodiversity and evolution. Looking ahead, LepEU is focused on training and mentoring the next generation of scientists, empowering them to integrate genomic analyses with phenotypic and ecological data to address ecological, evolutionary, and conservation questions.

The European Lepidopteran Population Genomics Consortium (LepEU, https://lepeu.github.io/organisation.html) is a community‐driven collaboration focused on using population genomics to study diverse species of Lepidoptera across Europe to address fundamental research in ecology and evolution, and to quantitatively inform biodiversity management strategies. Our first in‐person meeting, held in May 2025 in Montpellier, focused on coordinating efforts, building community, and setting future priorities and goals. Below, we outline the background and framework of LepEU, rationale for using Lepidoptera, a description of the consortium, achievements from the LepEU 2025 meeting, and future plans.

## 1 Background and Framework

Human activity is profoundly impacting biodiversity in diverse negative ways. Among the most alarming trends is the global decline in insect populations, which poses an existential threat to terrestrial ecosystem functioning and human food production. Reductions in insect abundance and diversity have been documented across various regions and taxonomic groups (e.g., Gebremariam 2024; Gross 2025; Hallmann et al. 2017; Lewinsohn et al. 2022; van der Sluijs 2020; van Klink et al. 2024; Wagner et al. 2021; Warren et al. 2021). These concerning trends are motivating scientific and political efforts to understand, monitor, protect, and restore biodiversity, and to inform coordinated, evidence‐based policy responses. Biodiversity genomics provides a unique set of information for population, species, and ecosystem management by estimating the spatial and temporal patterns of genetic variation across natural populations from local to continental scale, and by assessing how this variation connects with ecological resilience. Such insights can provide quantitative metrics of where conservation measures are meeting, exceeding, or lagging behind management goals, as well as when and where biological communities are likely to reach tipping points, where even rapid evolutionary responses will be unable to rescue them (e.g., Chevin and Bridle 2025).

Conservation genomics provides cost‐effective and quantitative insights into evolutionary processes, population structure, and demography (including connectivity, inbreeding, genetic load and effective population size) which are key parameters for assessing genetic health and adaptive potential (Willi et al. 2021). Accordingly, developing scalable genomic monitoring approaches has become a priority, as emphasized in the 2022 Strategic Plan of the Convention on Biological Diversity and the Kunming–Montreal Global Biodiversity Framework (www.cbd.int). These international frameworks call for policy‐relevant genomic indicators capable of tracking genetic diversity and its implications for population resilience (Hoban et al. 2024).

Although efforts to link population size estimates with genomic metrics are advancing, these relationships remain complex, even among well‐characterized taxa such as vertebrates (Yates et al. 2019; Wilder et al. 2023). For insects and other invertebrates, the connection between census size and commonly derived genomic parameters remains poorly understood. By extending genomic monitoring to these taxa, initiatives like LepEU will help close this critical knowledge gap and advance the development of inclusive, evolutionarily informed biodiversity metrics that are essential for forecasting species' responses to environmental change.

With these goals in focus, the Earth Biogenome Project aims to generate high‐quality reference genomes globally (e.g., Lewin et al. 2022; Blaxter et al. 2025). These reference genomes provide the foundation for resequencing projects to initiate genome‐informed studies of population and ecosystem dynamics. While biodiversity monitoring frameworks based on whole‐genome resequencing (WGS) data have been developed for selected species (e.g., Biodiversity Genomics Europe, https://biodiversitygenomics.eu/) and a growing number of additional projects are calling for similar frameworks, these have yet to be implemented across taxa within a comparative framework (e.g., Proposal for an EU Pollinator Monitoring Scheme; Potts et al. 2024). LepEU directly addresses these challenges by deploying biodiversity genomics across European Lepidoptera to establish a scalable framework for genomic monitoring.

## 2 The Rationale for Population Genomics of Butterflies and Moths in Europe

Lepidoptera, encompassing butterflies and moths, are excellent candidates for population genomics studies and align with the goal of understanding, conserving and managing biodiversity (e.g., Sucháčková Bartoňová et al. 2023). They represent a significant fraction of eukaryote diversity (10%–15%, Mora et al. 2011; Wiens 2023), with ~11,000 described species in Europe alone. In addition, moths and butterflies provide important ecosystem services (e.g., pollination; Requier et al. 2023), and include economically significant pests. Moreover, they typically capture the interest of the general public. Their positive public perception, rather unusual among insects, makes them an ideal flagship group for invertebrate studies and conservation efforts (Barua et al. 2012; van Tongeren et al. 2023).

Lepidoptera have long been used as indicators of ecosystem health, as well as of ongoing responses to environmental change (van Swaay et al. 2025). Moreover, Lepidoptera, especially butterflies, have a long history of well‐studied ecology and natural history (dating back to early naturalists and biogeographers), with this knowledge available in curated databases (e.g., LepTraits, Shirey et al. 2022). This allows a powerful integration between genomic insights and ecological traits, which is often unavailable for other clades. Importantly, many species can be reared under laboratory conditions, which is critical for certain genetic investigations and experimental manipulations. Furthermore, Lepidoptera offer a unique opportunity to connect demography (population size fluctuations) with population genomic measures, given the existence of exceptional long‐term, regional systematic monitoring programs (e.g., European Butterfly Monitoring Scheme, eBMS, https://butterfly‐monitoring.net/).

Lepidoptera are also an ideal group from a genomic perspective. Butterfly genome sizes are relatively small (~500 Mb; Liu et al. 2020; Wright et al. 2024) and can be readily assembled at high quality. This makes them a suitable clade for both reference genome sequencing initiatives and comparative population genomics studies. Over 1000 European species of Lepidoptera now have publicly available chromosome‐level reference genomes, thanks to collaborative efforts including the European node of the Earth Biogenome Project, the European Reference Genome Atlas (ERGA, https://www.erga‐biodiversity.eu/; Mc Cartney et al. 2024), the Darwin Tree of Life project (DToL, https://darwintreeoflife.org; The Darwin Tree of Life Project Consortium 2022), and, most notably, Project Psyche (https://projectpsyche.org; Wright et al. 2025). The latter aims to generate genomes for all ca. 11,000 species of Lepidoptera in Europe. This exceptional investment in lepidopteran genomes further reflects the strength of the active Lepidoptera research community, whose expertise spans molecular genetics, physiology, ecology, evolutionary biology, taxonomy, and conservation. Lepidoptera have also been extensively collected by amateur naturalists for over a century, creating rich museum collections that represent a largely untapped resource for reconstructing historical genetic and morphological variation.

## 3 The European Lepidopteran Population Genomics Consortium

Inspired by DrosEU, a consortium that leverages a shared research interest across Europe in population genomic studies of *Drosophila melanogaster* (https://droseu.net/, Kapun et al. 2021), we sought to gather a community focused on Lepidoptera. LepEU (https://lepeu.github.io) was established to study the population genomics of diverse species and communities of Lepidoptera across Europe. It aims to conduct comparative, continental‐scale genomic analyses of genetic diversity and evolutionary dynamics over space and time, while creating a baseline resource for research and biodiversity management.

**FIGURE 1:**
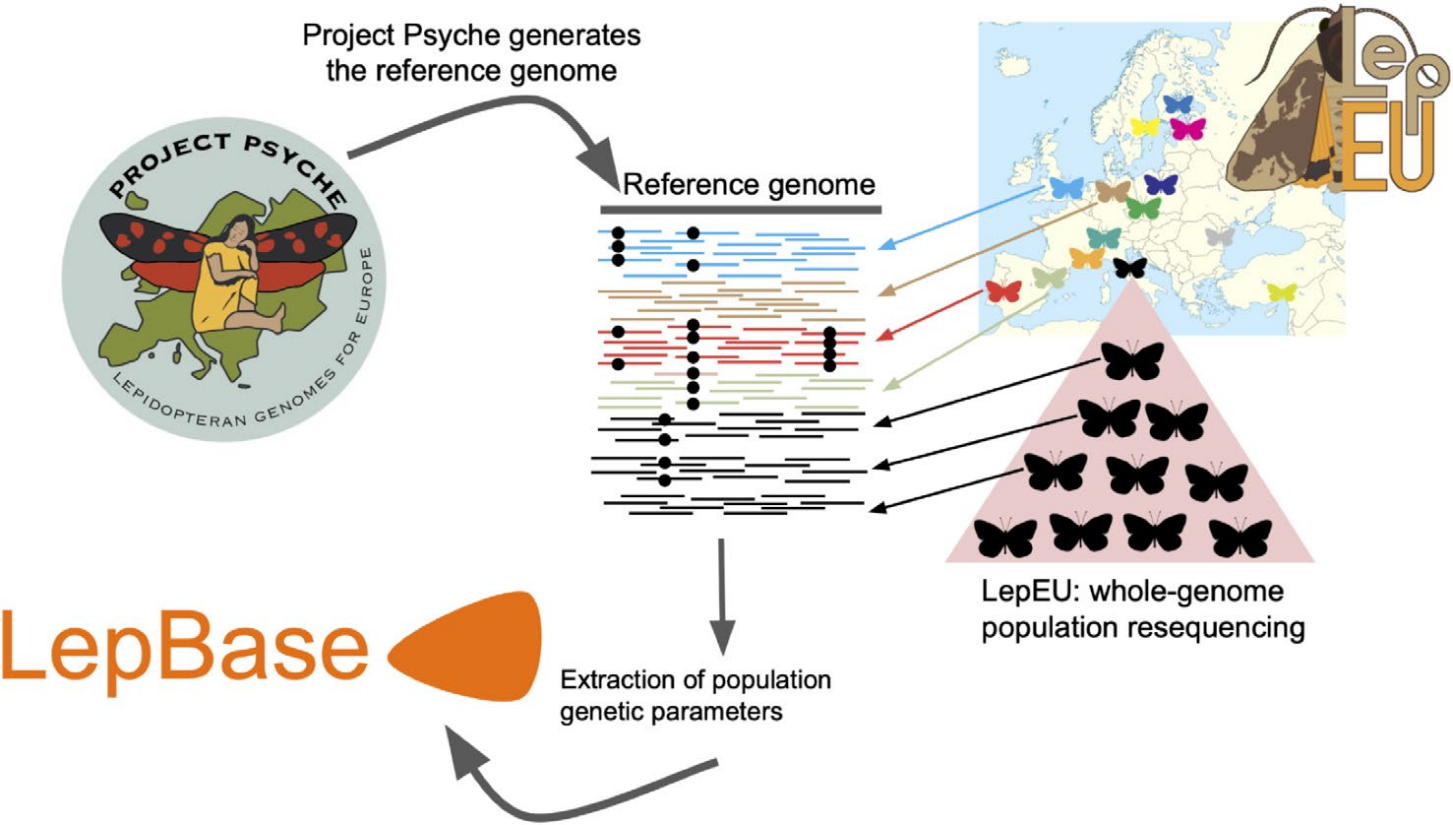
Project Psyche and LepEU complementary collaborative projects. Project Psyche is generating a chromosome‐level reference genome for each of the ca. 11,000 species of Lepidoptera in Europe. LepEU is producing short‐read population genomics datasets for a subset of these species from many locations across Europe. Multiple specimens are sequenced for each species at each location. The LepEU resequencing data is aligned to the reference genomes of Project Psyche. These alignments are analyzed to extract key population genetic parameters and these statistics are made openly available and interactively displayed in Lepbase (lepbase.org). Each location is indicated by a butterfly where the color represents a different sampling location. The short reads generated from individuals sequenced in each location are colored by the location they were collected in. Mutations are indicated by black dots. Map image credit: Alexrk2 (https://tr.wikipedia.org/wiki/Dosya:Europe_blank_laea_location_map.svg).

To achieve these aims, the LepEU network organizes field collections of population samples from various common and widespread species across Europe and facilitates their concerted whole genome resequencing. This strategy of focusing efforts on resequencing of individuals from diverse species is possible because of the exceptional growth in reference genomes produced by genomics consortia such as Project Psyche (Wright et al. 2025; Figure 1) and others.

**FIGURE 2:**
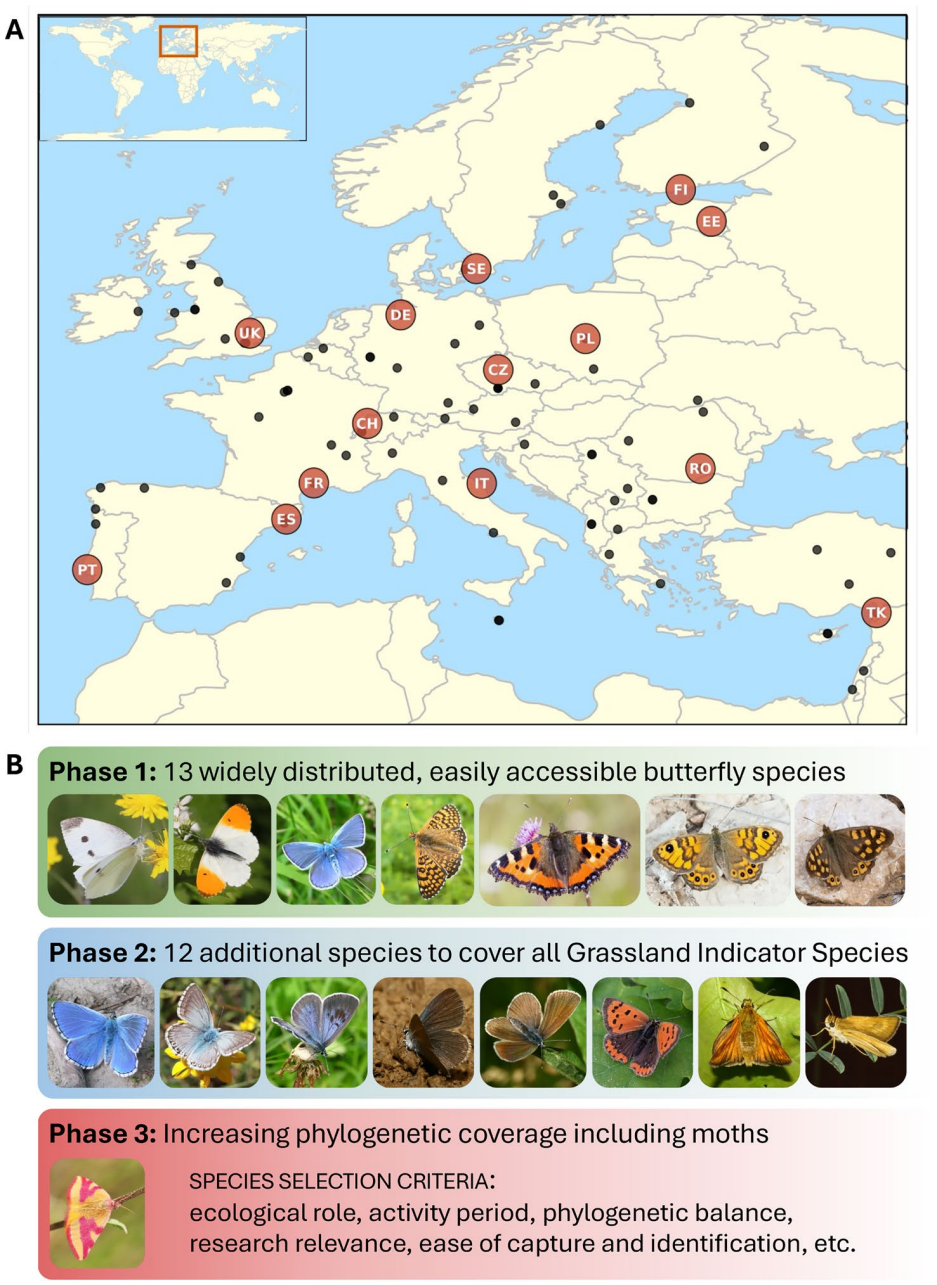
Sampling: Target sites and target species. (A) Map of Europe with LepEU participating regional coordinator locations (larger dots) as well as other member locations (smaller dots) who may provide specimens from different locations in each region. Updated sampling sites will be available on the LepEU website (https://lepeu.github.io). (B) Target species for each of the three stages (more information in Table S1). Images of some of the target species for Phase 1: *Pieris rapae* (AW), *Anthocharis cardamines* (MW), *Polyommatus icarus* (MW), *Melitaea cinxia* (MJ), *Aglais urticae* (VD), *Lasiommata megera* (VD), *Pararge aegeria* (RP). Phase 2: *Lysandra bellargus* (MW), *Lysandra coridon* (MW), *Phengaris arion* (MW), *Cupido minimus* (MW), *Cyaniris semiargus* (MW), *Lycaena phlaeas* (MW), *Ochlodes sylvanus* (MW), *Thymelicus acteon* (MW). Phase 3: *Lythria cruentaria* (AW). Photo credits: AW Arne Weinhold, MJ Mathieu Joron, MW Martin Wiemers, RP Riccardo Poloni, VD Vlad Dincǎ.

LepEU currently comprises collection efforts across 14 European countries, led by National Collection Coordinators and aided by over 100 researchers of diverse backgrounds and career stages (Figure 2A). After coordinating for over a year through virtual meetings, LepEU was able to hold its first in‐person meeting. Financial support was provided by the European Cooperation in Science and Technology (COST) Action “10kLepGenomes: Utilizing 10,000 genomes of European Lepidoptera” (www.10klepgenomes.eu), through its Working Group 4 (WG4). WG4 facilitates many of LepEU's goals (e.g., both organizations sharing similar leadership), by supporting the coordination of: (i) population‐level sampling, (ii) the generation of population genomic data, and (iii) comparative genomic analyses. The 10kLepGenomes Action also provides financial support enabling interactions among all member researchers (and especially early‐career researchers) to explore how emerging genomic resources can be used to tackle fundamental questions in evolutionary biology and to address pressing issues of biodiversity loss, pest control, and mitigating the effects of climate change.

## 4 The LepEU 2025 Meeting

Hosted at the Centre for Evolutionary and Functional Ecology (CNRS/University of Montpellier), the LepEU meeting focused on coordinating efforts, building community, and setting future priorities and goals. Working in person greatly facilitated making key decisions: setting immediate and longer‐term consortium goals, prioritizing sampling efforts, establishing standardized sampling protocols, and planning future work.

Standardized field collection efforts at the heart of LepEU are ongoing, organized by the network of National Collection Coordinators that manage regional specimen collections by local researchers, while ensuring that collection permits and Nagoya Protocol requirements are properly met (Figure 2A). By establishing this network, LepEU is able to coordinate species‐specific sampling across a diversity of ecosystems. Species collection efforts were formally organized into three phases reflecting the growth of the consortium (Figure 2B; Table S1). Phase 1 includes the initial group of 13 butterfly species selected based upon: (i) accessibility, (ii) broad continental distribution, (iii) ease of identification, (iii) role as biodiversity indicators, and (iv) availability of chromosome‐level reference genomes. These common species occupy (semi)natural habitats and include both ecological generalists and specialists. They pertain to three major butterfly families and vary in body size, dispersal ability, and host plant use, all of which are likely to influence regional patterns of genomic diversity. Phase 2 expands sampling to cover the remaining 12 of the 17 species used in the long‐running European Grassland Butterfly Indicator project (five species were already included in Phase 1), a standard measure of biodiversity in Europe (van Swaay et al. 2025). By gathering regional samples of all European Grassland Butterfly Indicator species, we aim to provide a valuable resource for integrating decades of regional observational data with genomic insights, allowing the development of meaningful genetic metrics that capture observed demographic trends. Phase 3 expands the sampling scope to include moth species, to broaden phylogenetic and ecological coverage with a focus on contributing to the EU Pollinators Initiative (Potts et al. 2024). The exact number and identity of Phase 3 species are yet to be defined; they will be selected according to similar criteria as Phase 1 species (accessibility, geographic distribution, reliability of identification, phylogenetic coverage), while also considering research interest, funding opportunities, and public outreach.

**FIGURE 3:**
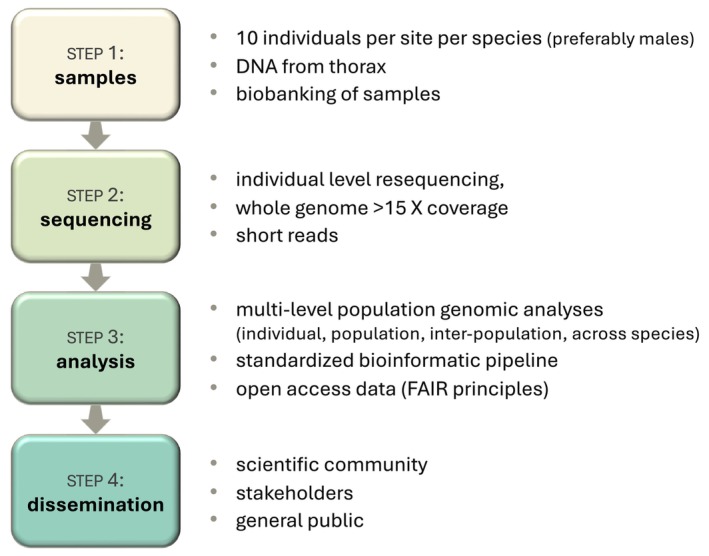
LepEU workflow in four steps. Additional protocol details are available on the LepEU website (https://lepeu.github.io/protocols.html).

The LepEU workflow includes four main steps (Figure 3): (i) field sampling, storage, and DNA isolation, (ii) individual‐level short‐read WGS, (iii) data analysis, (iv) communication, and dissemination. Field sampling and storage is organized nationally by National Collection Coordinators, while DNA extractions will take place in labs across Europe with dedicated high‐throughput equipment. During the workshop, attendees brought a subset of field collections for DNA extraction, comprising approximately 500 specimens representing diverse species and sampling locations. This activity not only facilitated the training of early‐career researchers in laboratory work, but it also allowed a quantitative assessment of the minimal standards of sampling handling and processing. Beyond agreeing upon protocols for collection and preparation (https://lepeu.github.io/protocols.html), a database management scheme was developed for tracking sample metadata through our sample pipeline. Our database promotes standardization and data interoperability with other biodiversity initiatives, such as Project Psyche, DToL, ERGA, and the Global Biodiversity Information Facility (GBIF 2020).

DNA samples meeting minimum quality and quantity criteria (see LepEU website protocols page) are being processed for genome sequencing, using Illumina WGS targeting a minimum of 15X genome coverage per individual. The resulting genomic data allow the quantification of genetic diversity metrics at the level of individuals and populations relevant to global biodiversity monitoring initiatives (e.g., heterozygosity, nucleotide diversity, effective population size, inbreeding values, population structure and history), as well as the identification of the genomic features associated with signatures of spatial and temporal adaptation. We will integrate gene annotation information when required by downstream analyses. Most Phase 1 species have available chromosome‐level reference annotations (Table S1), so we will use those resources directly. When suitable annotations are lacking, we will apply a standardized, evidence‐based annotation strategy (leveraging homology‐ and transcript‐informed approaches where available) to define comparable genomic partitions across species. This will enable analyses that differentiate coding and noncoding regions and, where robustly inferred, putatively neutral site classes (e.g., four‐fold degenerate sites), thereby improving comparability and interpretation of population‐genetic inferences across the consortium dataset. Analyses will be conducted using consortium‐developed bioinformatics pipelines, which will be openly shared via the Wellcome Sanger Institute and the Project Psyche via an updated version of the lepidopteran genome database, Lepbase (Challis et al. 2016), as well as general public repositories, such as the European Nucleotide Archive (ENA, https://www.ebi.ac.uk/ena/browser/home). To foster capacity building within our community, LepEU will run training schools focusing on WGS data analyses (e.g., comparative population genomics), through COST support. One important near‐term objective will be the development of best‐practice analysis pipelines that perform reliably across the phylogenetic depth of sampled taxa. This may be challenging, as Lepidoptera span more than 230 million years (My) of evolution (Fiorelli et al. 2025; Kawahara et al. 2019; Wahlberg et al. 2013), with the greatest diversity being in the approximately 120 My old clade Ditrysia (Kawahara et al. 2019; Wahlberg et al. 2013).

Participants formalized plans for dissemination of results, including peer‐reviewed publications, policy briefs, and public outreach. Guidelines on authorship and data use were defined, with emphasis on inclusiveness and community spirit, under the understanding that corresponding documentation will be shared and updated on the website (see LepEU website protocols page). Sequencing data, pipelines, and results will be made available to the broader scientific community and policy stakeholders. The consortium highlighted the importance of strengthening engagement with external actors, including policymakers and local communities. We discussed how to integrate with Working Group 1 of the 10KLepGenomes COST action, which is dedicated to making connections between relevant conservation and industry stakeholders. This will be greatly facilitated by existing LepEU members direct involvement with WG1.

Securing dedicated funding to support sampling, sequencing, and analysis is essential to the success of LepEU. Community‐building activities for the next 3 years is provided by the COST Action 10kLepGenomes for networking, collections, coordination, and training. Field collections, data generation, and data analysis are, however, underfunded. To date, field sampling has been organised using local resources and on a volunteer basis from participating laboratories. DNA isolation and sequencing require more substantial resources. Several LepEU member laboratories have allocated existing resources to initiate DNA isolation and sequencing on a subset of species (Table S1). Several national‐level grants have already been obtained (e.g., see Funding section), with several others pending that will support LepEU goals.

Sequencing for Phase 1 is facilitated by the Wellcome Sanger Institute, UK, for samples collected in the UK, Switzerland, and Italy, and other national level funding support. All raw sequencing data will be made available through INSDC databases (https://www.insdc.org/). The Wellcome Sanger Institute has also offered to host the population genomic data and results generated by LepEU following the FAIR principles (Wellcome Sanger Institute 2025). Our first meeting generated a strong, shared commitment to securing sustained support for sampling, sequencing, and analysis, and a broad consensus on the need to secure large‐scale funding at the EU level. Furthermore, LepEU members agreed on proceeding with a pilot analysis of a limited number of species using available funds (Table S1), that will serve as a basis for analysis pipeline development and a proof of concept for scaling up to the multispecies goals envisioned by LepEU.

## 5 Overview and Outlook

LepEU is a collaborative international consortium aimed at generating population‐level genomic data on a continental scale for widespread European Lepidoptera. We are focused on assessing genetic diversity within and among populations, understanding population history and the evolutionary forces that shape them, identifying connectivity patterns, and detecting genetic diversity reservoirs that could help reverse genetic erosion trends. These insights will provide a robust empirical dataset useful for multiple aims, including contributing to conservation guidelines and policy recommendations, developing genomically informed conservation management strategies, as well as addressing fundamental questions in evolutionary biology, spanning topics from phylogeography to the comparative analysis of regional adaptation.

By fostering collaboration across Europe, LepEU transcends the limitations of individual research groups and provides a continental‐scale genomic perspective on lepidopteran biodiversity. It establishes a strong foundation for long‐term, collaborative research by generating genomic data that supports census‐based assessments of lepidopteran diversity (e.g., eBMS). In doing so, LepEU contributes pioneering genomic data from diverse Lepidoptera for a deeper understanding of insect biodiversity that will promote the integration of genomics with census‐based data for developing the next generation of biodiversity monitoring tools. Moreover, the fact that LepEU is a consortium will facilitate knowledge transfer and capacity building among members and the broader community.

While our initial focus prioritizes wide regional sampling of common species, LepEU's data generation and community building will facilitate more ambitious research directions, including: (1) exploring other geographic dimensions, such as altitudinal clines, urban gradients, narrowly distributed or regionally threatened species, and comparisons among specific population pairs; (2) examining relationships between species, their larval host plants, and associated microbiota across their ranges; (3) incorporating temporal replication by sampling the same populations across years or across seasons to detect rapid evolutionary dynamics; (4) leveraging archived museum specimens to assess historical trends in genetic diversity under anthropogenic pressures; and (5) using additional phenotypes from collected specimens (e.g., size, pigmentation, gene expression data). Together, these directions move LepEU from descriptive population surveys toward a hypothesis‐driven framework for testing spatial evolutionary theory. Theoretical models and empirical studies highlight that gene flow can both expand and constrain local limits, emphasizing the intricate interplay among selection, demography, and population structure across a species' geographical and ecological range (Bridle and Hoffmann 2022). By integrating these perspectives, LepEU aims to generate a dynamic, multidimensional view of European butterfly genomics and biodiversity—capitalizing on detailed ecological and historical knowledge to test fundamental theory on evolutionary responses to environmental change and their consequences for ecological resilience.

## Funding

This work was supported by European Cooperation in Science and Technology Cost Action CA23122: Utilizing 10,000 genomes of European Lepidoptera (CA23122 10kLepGenomes; https://www.cost.eu/actions/CA23122/); Agence Nationale de la Recherche (ANR) and AAPMUSE‐PhenoLep of the University of Montpellier (ANR‐20‐CE02‐0017 to MJ); Carl Tryggers Foundation, SE (CTS 24: 3554 to CWW); Czech Ministry of Education, Youth and Sports through the sub‐programme INTER‐COST (grant no. LUC25115 to PMM); LMUmentoring and LMUexcellent Support Funds (to AW); Jane & Aatos Erkko Foundation (to MS); Swedish Research Council (2022–04507 to CWW); Swiss National Science Foundation (202,869, 220,868 to KL); Wellcome Trust (220,540/Z/20/A and grant no. 218328 to MB, JIM, CJW).

## Conflicts of Interest

The authors declare no conflicts of interest.

## Supporting information


**Table S1:** Target species.

## Data Availability

No data were used to support this study.
