# Supplementary material for: LepEU: A Consortium to Study the Population Genomics of Butterflies and Moths Across Europe
Source: Evol Appl. 2026 May 12;19(5):e70246. doi: 10.1111/eva.70246 (PMC13161756; doi:10.1111/eva.70246)
Supplement: Supplementary file 1 — Table S1: Target species. [file EVA-19-e70246-s001.docx]

### Table S1: target species

Phase 1: wide-range of distribution and easily accessible

| **Scientific name** | **Common name** | **Family/subfamily** | **Genbank assembly** |
| --- | --- | --- | --- |
| *Gonepteryx rhamni* (Linnaeus, 1758) | Brimstone | Pieridae/Coliadinae | GCA_965217435.1 |
| *Anthocharis cardamines* (Linnaeus, 1758) | Orange tip | Pieridae/Pierinae | GCA_905404175.1 |
| *Pieris napi*  (Linnaeus, 1758) | Green-veined white | Pieridae/Pierinae | GCA_905475465.2 (RefSeq annotated) |
| *Pieris rapae*  (Linnaeus, 1758) | Small cabbage white | Pieridae/Pierinae | GCA_905147795 (RefSeq annotated) |
| *Callophrys rubi*  (Linnaeus, 1758) | Green hairstreak | Lycaenidae/Theclinae |  |
| *Polyommatus icarus* (Rottemburg, 1775) | Common blue | Lycaenidae/Polyommatinae | GCA_937595015.1 |
| *Coenonympha pamphilus* (Linnaeus, 1758) | Small heath | Nymphalidae/Satyrinae |  |
| *Lasiommata megera* (Linnaeus, 1767) | Wall brown | Nymphalidae/Satyrinae | GCA_928268935.1 |
| *Pararge aegeria*  (Linnaeus, 1758) | Speckled wood | Nymphalidae/Satyrinae | GCA_905163445.1 (RefSeq annotated) |
| *Maniola jurtina*  (Linnaeus, 1758) | Meadow brown | Nymphalidae/Satyrinae | GCA_905333055.1 (RefSeq annotated) |
| *Melitaea cinxia*  (Linnaeus, 1758) | Glanville fritillary | Nymphalidae/Nymphalinae | GCA_905220565.1 (RefSeq annotated) |
| *Aglais io*  (Linnaeus, 1758) | Peacock butterfly | Nymphalidae/Nymphalinae | GCA_905147045.1 (RefSeq annotated) |
| *Aglais urticae*  (Linnaeus, 1758) | Small tortoiseshell | Nymphalidae/Nymphalinae | GCA_905147175.2 |

Phase 2: extend list to cover all grassland indicator species

| **Scientific name** | **Common name** | **Family/subfamily** | **Genbank assembly** |
| --- | --- | --- | --- |
| *Ochlodes sylvanus*  (Esper, 1777) | Large skipper | Hesperiidae/Hesperiinae | GCA_905404295.2 |
| *Thymelicus acteon* (Rottemburg, 1775) | Lulworth skipper | Hesperiidae/Hesperiinae | GCA_951805285.1 |
| *Erynnis tages*  (Linnaeus, 1758) | Dingy skipper | Hesperiidae/Pyrginae | GCA_905147235.1 |
| *Spialia sertorius* (Hoffmannsegg, 1804) | Red-underwing skipper | Hesperiidae/Pyrginae | GCA_964258965.1 |
| *Lycaena phlaeas*  (Linnaeus, 1761) | Small copper | Lycaenidae/Lycaeninae | GCA_905333005.2 |
| *Euphydryas aurinia* (Rottemburg, 1775) | Marsh fritillary | Nymphalidae/Nymphalinae | GCA_965226145.1 |
| *Cupido minimus*  (Fuessly, 1775) | Small blue | Lycaenidae/Polyommatinae | GCA_965195375.1 |
| *Cyaniris semiargus* (Rottemburg, 1775) | Mazarine blue | Lycaenidae/Polyommatinae | GCA_905187585.1 |
| *Phengaris arion*  (Linnaeus, 1758) | Large blue | Lycaenidae/Polyommatinae | GCA_963565745.1 |
| *Phengaris nausithous* (Bergsträsser, 1779) | Dusky large blue | Lycaenidae/Polyommatinae |  |
| *Lysandra bellargus* (Rottemburg, 1775) | Adonis blue | Lycaenidae/Polyommatinae | GCA_905333045.1 |
| *Lysandra coridon*  (Poda, 1761) | Chalkhill blue | Lycaenidae/Polyommatinae | GCA_905220515.1 |

Phase 3: extend list to include moths (and contribute to pollinator initiative)

Although specific species have not yet been identified, selection will be guided by criteria such as ecological role (e.g., pest or pollinator status), activity period (e.g., night-flying / day-flying species), phylogenetic representation, relevance as research models, and practical considerations including ease of capture and identification.
